# Decoding the Role of Gut-Microbiome in the Food Addiction Paradigm

**DOI:** 10.3390/ijerph18136825

**Published:** 2021-06-25

**Authors:** Marta G. Novelle

**Affiliations:** 1 Center for Research in Molecular Medicine and Chronic Diseases, CIMUS, University of Santiago de Compostela-Instituto de Investigación Sanitaria (IDIS), 15782 Santiago de Compostela, Spain; marta.garrido@usc.es; 2 CIBER Fisiopatología de la Obesidad y Nutrición (CIBERobn), Instituto de Salud Carlos III, 28029 Madrid, Spain

**Keywords:** food addiction, eating behaviour, reward, obesity, gut-microbiome, gut-dysbiosis

## Abstract

Eating behaviour is characterised by a solid balance between homeostatic and hedonic regulatory mechanisms at the central level and highly influenced by peripheral signals. Among these signals, those generated by the gut microbiota have achieved relevance in recent years. Despite this complex regulation, under certain circumstances eating behaviour can be deregulated becoming addictive. Although there is still an ongoing debate about the food addiction concept, studies agree that patients with eating addictive behaviour present similar symptoms to those experienced by drug addicts, by affecting central areas involved in the control of motivated behaviour. In this context, this review tries to summarise the main data regarding the role of the gut microbiome in eating behaviour and how a gut dysbiosis can be responsible for a maladaptive behaviour such as “food addiction”.

*“All disease begins in the gut.”*

Hippocrates

## 1. Introduction

Despite wide daily variation in food intake and energy expenditure, in most subjects body weight remains constant over long periods of time, due to a continuous regulation of both processes. This regulation is tightly controlled through effects on the energy store, integrated by the central nervous system (CNS) and modulated by endocrine and nervous signals from peripheral organs [1]. However, this homeostatic pathway can be neutralized by a more flexible non-homeostatic pathway. In fact, external cues, cognitive and emotional factors can override the homeostatic process and finally the motivational and reward pathways become crucial in the regulation of eating behaviour, hence food intake process goes beyond metabolic needs [2,3,4]. If this deregulated situation is maintained over time, it can lead to complicated and addictive behaviors, such as the behavioral addiction to eating [5,6]. In this context, the gut, with its own nervous system, the enteric nervous system (ENS), is considered as a second brain due to its direct communication network with the CNS and plays a key role by regulating both homeostatic and non-homeostatic responses. This network integrates gut signals and links them, mainly through the vagus nerve, to cognitive and reward centres of the brain, therefore modulating behavioral responses [7]. Furthermore, over recent years, an interesting new actor has emerged in this equation, hence we can talk now about the “gut–microbiota–brain (GMB) axis” [8,9]. The gut microbiota (GM) is composed of a complex and dynamic population of microorganisms that offer many benefits through their close interaction with the host. These symbiotic microorganisms are not only essential for the fundamental physiological functions and to maintain gastrointestinal (GI) homeostasis, but a growing body of evidence supports that this “superorganism” may also interact with the host neuroendocrine system and modify brain development and responses, resulting in modifications of the host behaviour [10,11,12]. Similarly, gut microbiota could be a stressor target in maladaptive behaviors. In fact, environmental, physical and psychological stress present in daily life has been linked to gut dysbiosis. Adding support to this contention, both in animal and human models it has been demonstrated that the manipulation of GM alters levels of stress hormones and the ingestion of specific probiotics could rectify some of the abnormalities observed. Hence, a healthy bidirectional communication system between GM and the CNS is an essential element to prevent psychological disorders [13]. Noteworthy, evidence emerging from both human and animal studies proposes that GM has essentially contributed to current cognitive development and human social behaviour [14,15]. Because of the vast body of literature respecting this topic, the main focus of this mini-narrative review will be to provide a concise overview of the current research regarding the interrelationship between gut microbiome dysbiosis and dysfunctional eating behaviour, such as food eating addiction. In order to contextualize their relevance, these aspects will be preceded by a brief summary of the physiological role of the non-homeostatic pathways and the GM in the regulation of food intake and eating behaviour. Finally, future directions will be discussed. Decoding in depth the role of GM in food addiction could provide promising opportunities for future therapeutic options.

## 2. Non-Homeostatic Contribution to Regulation of Food Intake

As has been mentioned above, food intake behaviour is a highly regulated process by many redundant mechanisms. This regulation is the result of the integration of two neuronal circuits that overlap both anatomically and functionally: the homeostatic pathway, which controls the energy balance by triggering food intake in response to a depletion of energy stores, and the more flexible non-homeostatic one, hedonic or reward-based, which is driven by pleasurable emotions and previously learned behaviors. Current data based on opto- and chemo-genetic studies conducted in animal models, support that these two key systems are part of a more complex motivational system and both cannot be functionally dissociable from one another [5,16]. In this context, the lateral hypothalamic area (LHA) is a crucial area integrating homeostatic and reward-related central and peripheral signals and coordinating adaptative behavioral responses to the nutritional background. Hence, the LHA serves as a reward–motivation–cognition hub. Interestingly, in addition to being implicated in feeding behaviors, LHA regulates sleep/wake states and arousal, so finally the decision to eat can be also modulated by circadian time [17,18,19,20]. However, due to the impact of the present food environment, the hedonic pathway is continuously tested and overwhelmed by countless signals contributing to a deregulated circuit and finally to the development of eating disorders.

Historically, reward-based regulation is comprised of three different aspects: “liking”, “wanting” and “learning”, that despite being highly connected can be disassociated anatomically and manipulated in animal models to obtain behaviors that are either exclusively pleasant or motivational in response to a food stimulus. Hence, while “liking” is closer to sensory processes, “wanting” is closer to decision making and goal-seeking, by reflecting the cue-driven tendency to choose one behaviour rather than another to optimize rewards. One decisive mechanism for disordered eating is the progression from normal “liking” and “wanting” to addictive behaviour. On the other hand, the “learning” process includes associations with anticipation of rewards [21,22]. In fact, data obtained on animal models suggest that the repercussions of food intake can be predicted and accommodated in response to external food cues based on previous experiences [23,24]. In this regard, efferent information through the visual, olfactory, auditory and oral taste systems is integrated with large areas of the brain, constituting our food memories that will modulate future eating behaviors [2]. Hence, in the course of time, animals not only undergo rewards, but also anticipate them [25]. This cue-induced anticipation can lead to devaluation of recompense value; however, a malfunctioning of such behaviour may propitiate an unsuitable responding to food cues and dysregulation of food intake [26]. In this sense, recent data, obtained from animal models, have demonstrated that chronic consumption of refined, high-fat or high-sugar diets can cause persistent aberrations in behavioral control resulting in insensitivity to food devaluation and increasing impulsive decision and cravings [27,28]. In agreement, it has been proposed that overeating observed in obese humans can be due to a lower adaptation of eating behaviour with respect to changes in motivational value. In consequence, obese humans exhibit maladaptive behaviors such as eating in the absence of hunger, or present late meal cessation [29].

On the other hand, many rewards act as instrumental reinforcers determined by the balance between goal-directed (or “conscious”) and habitual (or “automatic”) processes [30]. Usually, high-value reinforcements, such as palatable food, can cause a pathological imbalance of these behaviors. In fact, these positive stimuli are known to bias behaviour toward the “habit system” and to accelerate habit formation [31,32]. Thence, food intake will be initiated without previous wanting and this action will not be finished even if the motivational value has already decreased [33]. Although, initially, habits can be helpful and adaptative in daily life, these may result in becoming rigid and inflexible. Therefore, the lack of the ability to shift back to cognitive behaviour will cause some habits to become obsessive and compulsive under different circumstances, such as stress and anxiety [33,34,35]. Taking all these data together, it is easier to understand why a positive correlation between anxiety and food addiction is normally observed. Indeed, the emotion dysregulation theory, which comprises the inability to flexibly respond to and direct emotions, has been proposed as a predictor of food addiction [36]. Moreover, uncontrolled emotional eating can be a coping mechanism to regulate distress, despite the negative consequences [37,38]. Finally, to highlight similarities with other natural rewards and with drug abuse, a maladaptive reinforcer devaluation process can evoke an aversive state when having to wait for access to the liked/wanted reward. Hence, while in the first instance a binge eating behaviour is positively reinforced, the palatable food ultimately acquires negative reinforcing properties [25].

The neuro-regulation of these processes is based on a complex network, which, according to a great deal of evidence, shares the same brain pathways as other addictive substances and behaviors [39,40]. Non-homeostatic factors are mainly processed in corticolimbic structures such as the prefrontal cortex (PFC), predominantly within the orbitofrontal cortex (OFC) projections, the amygdala, nucleus accumbens (NAc) and ventral tegmental area (VTA), where opioids and dopamine (DA), together with other neurotransmitters, are released promoting the sensation of pleasure and incentive salience, respectively. Hence, the dopamine striatal system is mainly (although not exclusively) involved in “wanting”, and the opioid and cannabinoid systems are mainly (although not exclusively) involved in food “liking” [41]. Although the mesolimbic dopamine pathway from the VTA to the striatum has been perhaps the most strongly linked to reward, over recent years, the PFC areas have gained functional relevance due to their role in focusing attention, controlling motivation and assigning reward value. Indeed, PFC acts as a top-down mechanism to suppress the bottom-up drives, such as impulsivity coming from the ventral striatum and compulsivity from the dorsal striatum [42]. To be noted, within prefrontal circuitries, two opposing systems have been described; one which motivates craving and involves habits, a “GO system”, specifically the prelimbic cortex (PL), and one which instead inhibits these behaviors by suppressing emotional responses to stimuli, a “STOP system”, located in the infralimbic cortex (IL) [43,44]. An imbalance of both systems with increased responsiveness to food cues could explain the abnormal activation of PFC regions observed in drug/food addicted humans [45].

On the other hand, metabolic signals from the periphery can also modulate all levels of food-related cognitive and reward processing. In this context, adipocyte-derived hormones, such as leptin, exert a wide influence across many reward-implicated brain regions by modulating midbrain dopamine and opioidergic pathways and finally suppressing the incentive value of food [46,47,48]. Likewise, GI hormones, acting either directly from the bloodstream or via the vagus nerve, have a great impact on eating behaviour [17]. In this sense, ghrelin, the only known peripherally-derived orexigenic peptide hormone secreted from the stomach, also reinforces food reward, in part through amplification of dopaminergic signaling mechanisms [49]. Contrarily, many anorexigenic enteroendocrine signals, such as peptide YY (PYY) and glucagon-like peptide-1 (GLP-1), suppress reward function and can increase anxiety-like behaviour [50]. Finally, the influence of gonadal hormones, such as estrogens, should be remarked in energy homeostasis, both in homeostatic and non-homeostatic components. Knowing the function of estrogens in eating behaviour can help us to understand why women are more vulnerable to eating disorder (ED) symptoms. Indeed, ED prevalence is the most solid sex difference in all psychiatric pathologies, with women being up to ten times as likely as men to suffer from an ED [51,52].

**Figure 1:**
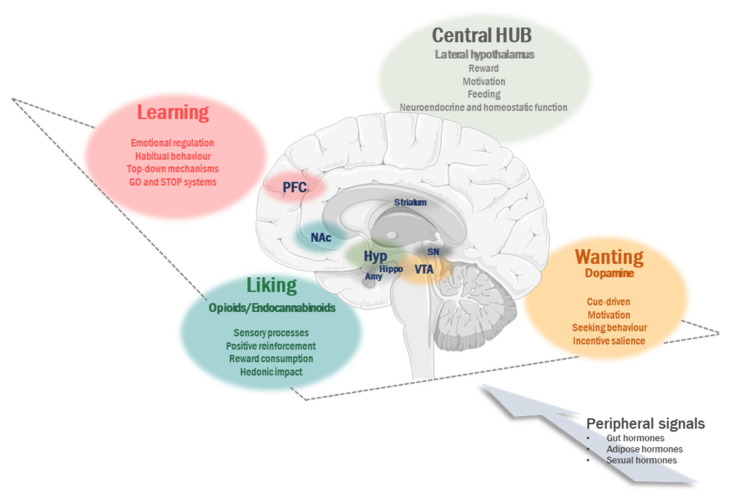
Non-homeostatic aspects implicated in the regulation of eating behaviour. The reward-based regulation is comprised of three different aspects: “liking”, “wanting” and “learning” that can be disassociated anatomically in different brain areas. Amy, amygdala; Hippo, hippocampus; Hyp, hypothalamus; Nac, nucleus accumbens; PFC, prefrontal cortex; SN, substantia nigra; VTA, ventral tegmental area. All these areas can be modulated by different peripheral signals.

As previously recorded, reviewing in depth the neuro-regulation of the non-homeostatic component of food intake and eating behaviour is complex and beyond the aim of this paper, but detailed manuscripts on this topic can be found elsewhere [5,16,17,52,53,54] (Figure 1).

## 3. Gut Microbiota: A Key Player in the Regulation of Eating Behaviour

**Figure 2:**
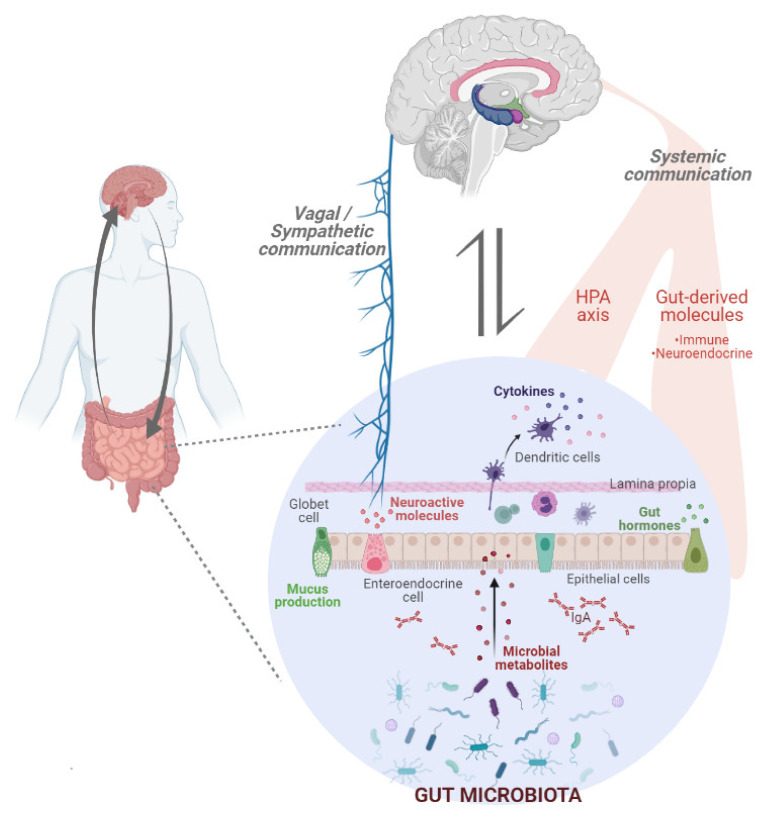
Bidirectional communication network between the Gut and the Central Nervous System (CNS). Gut microbiota and microbial metabolites are able to regulate the host energy metabolism and host eating behaviour by acting on the CNS through different pathways. On the other hand, CNS modulates microbiota composition and function.

Supported by abundant research, it is well-known that a sophisticated bidirectional communication network exists between the gut and the CNS. This gut–brain axis, with high influence on behaviour and other basic CNS functions, is composed of the cited CNS, the ENS, the sympathetic and parasympathetic branches of the autonomic nervous system (ANS) and the hypothalamic–pituitary–adrenal (HPA) axis [9,55,56] (Figure 2). In addition, over recent decades, a new decisive player has emerged in this equation [57]. Living gut microorganisms and their bioactive metabolites, including short-chain fatty acids (SCFAs) and conjugated fatty acids among others, and their neuroactive metabolites, such as serotonin (5-hydroxytryptamine; 5-HT) and γ-aminobutyric acid (GABA), are released in the bloodstream and can modulate the CNS directly or indirectly by affecting the ANS [58,59,60]. Hence, changes in enteric neuron activity will be perceived by the vagus nerve to modulate, among other physiological aspects, appetite, satiety, stress, and mood. This fact is in accordance with data obtained from rodent models. In this sense, ingestion of probiotics, such as *Bifidobacterium longum NCC3001*, induces activation of the vagal sensory neurons that innervate the GI enhancing exploratory behaviour in healthy mice [61]. However, mice that have undergone vagotomy did not show the same positive changes in emotional behaviour after probiotics ingestion, as was also observed in intact animals after *Lactobacillus rhamnosus JB-1* treatment [62]. In agreement, promising results from clinical trials on humans show that probiotic ingestion is associated with changes in the activity of multiple brain areas involved in emotional processing, including the amygdala and fronto-limbic regions [63]. In addition, to interact with the vagus nerve, some bacterial strains can also influence gut hormone secretion, including PYY, GLP-1, leptin, and ghrelin, and thus affect appetite and satiety via hypothalamic neuroendocrine pathways [64,65,66].

Likewise, since the GI tract comprises the large junction between the microorganisms and the immune system, GM is able to modulate this, as well as cytokine production [67]. These cytokines will also be released and act on the CNS, provoking changes in host behaviour, especially related to social conduct. In fact, inflammatory activity can alter cognition and motivation, having important consequences on eating behaviour [68]. In this context, it has been described that inflammatory mediators can act on cortico-amygdala threat and cortico-basal ganglia reward circuitries, predisposing individuals to addictive habits and increasing consumption of highly palatable diets [69]. In addition, GM also regulates microglial maturation and function [70,71]. Interestingly, microglia, the major player in brain inflammation, is essential for the development and preservation of addictive behaviors by influencing neuronal and synaptic functions in diverse ways [72]. Indeed, Gutiérrez-Marcos et al. demonstrated that microglia activation and neuroinflammatory processes induced overeating behaviour in mice. The access to highly palatable food led to overconsumption and caused functional alterations in the reward system, especially in the NAc [73]. Besides, in inflammatory states, overactivation and dysregulation of microglia can have important consequences on blood–brain barrier (BBB) integrity [74].

Finally, we highlight that GM can itself be considered as a “virtual endocrine organ” [75]. GM and the factors that it produces interact with the host endocrine system, disturbing both brain function and eating behaviour [76]. Moreover, GM can affect the postnatal development of the stress response [77] and exacerbate this response via the HPA-axis [78] as observed in pre-clinical models. It has been previously mentioned that stress is a crucial factor in eating behaviour by regulating food preference and it has also been described as the common factor behind some eating-associated disorders [79,80]. Likewise, food cues and a stressful environment can promote food craving and intake by increasing total ghrelin and cortisol levels [81]. Hence, changes in stress-related neurohormones, such as norepinephrine and epinephrine, could be one of the mechanisms through which GM modulates food intake.

On the other hand, the brain has a prominent role in the modulation of gut activities. Indeed, it can affect microbiota composition and function by alteration of intestinal permeability and by stimulating the immune response [82]. Hence, interruption of these bidirectional interactions and changes in the microbial environment can be involved in the pathogenic pathways responsible for the development of CNS disorders, as has been demonstrated by different studies. In fact, neuropsychiatric comorbidity is a common finding in patients with a functional GI disorder and/or gut dysbiosis [55,83,84,85]. Consequently, microbiome-based strategies, including prebiotics, probiotics and fecal transplants, as well as dietary changes, have been proposed as new therapeutic treatments to improve mental health [86,87,88]. Indeed, some probiotics capable of producing neuroactive substances have been described as psycho-biotics due to their potential to act as psychotropic agents [89].

The GM is constituted by a huge population of microorganisms, consisting predominantly of different phyla of bacteria, and a small number of viruses, protozoa and fungi. Although the microbiota composition in the GI is reflective of life events such as illness, antibiotic treatment and mainly dietary habits, being modulated over time, a “healthy gut microbiota” is characterised by the presence of *Firmicutes* and *Bacteroidetes*, as well as *Actinobacteria*, *Proteobacteria*, *Fusobacteria* and *Verrumicrobia* phyla in relatively low amounts [11,90]. As already mentioned, gut microorganisms provide a wide variety of beneficial functions to the host as a result of their own metabolism.

Under anaerobic conditions, undigested carbohydrates are fermented mostly into SCFAs, such as acetate, propionate and butyrate. These molecules have multiple effects, principally on host metabolism [91]. Noteworthily, both preclinical studies [92,93,94] and clinical studies [95] have demonstrated that dietary supplementation of SCFAs protect from metabolic disorders, such as obesity [96]. In this context, an important work conducted by De Vadder et al. showed that these microbiota metabolites are able to induce intestinal gluconeogenesis via different mechanisms and finally to promote metabolic benefits, such as reduced body weight and improved insulin sensitivity, etc., through the activation of specific brain targets, some implicated in appetite control [97]. Accordingly, gut dysbiosis, which causes disruption of SCFA metabolism, can promote hyperinsulinemia and potentially increase hedonic intake [98]. In agreement with these data, other studies also highlight the important role of SCFA in eating regulation [99]. In fact, this has been demonstrated in animal models exposed to an HFD and highly fermentable carbohydrate (FC), as the acetate derived from the colon was able to induce an anorectic signal in the hypothalamus [100]. In contrast, Perry et al. described how in HFD-fed rats acetate led to increased ghrelin levels and glucose-stimulated insulin secretion that ultimately promoted hyperphagia [101]. Hence, although there is clear evidence regarding the interrelation of acetate and central effects on appetite regulation, other aspects and apparent discrepancies should still be covered in-depth [102]. On the other hand, in animal models, both butyrate and propionate have also been proposed as intermediate signals regulating food intake that exert their anorectic effect by modulating gut hormone release [93,103]. Analogous results were observed in human models. Indeed, propionate significantly stimulated the release of anorexigenic hormones PYY and GLP1 from human colonic cells. In agreement, Chambers et al. observed that, after acute delivery of propionate, specifically to the colon, healthy subjects showed an increase in plasma levels and PYY and GLP-1 levels, and consequently inhibition of energy intake [104]. Therefore, Torres-Fuentes et al. showed how SCFAs and other microbiota metabolites can attenuate ghrelin receptor signaling [65]. In addition, propionate is able to attenuate the reward effects on feeding behaviors through a reduction in anticipatory response to high-energy foods via the striatal pathway, decreasing caudate and NAc activity [105]. Similar results were found by Li et al. in a well-conducted work. These authors demonstrated how butyrate administration reduced appetite, activated thermogenesis and improved lipid and glucose metabolism via vagal nerve signaling in mice [106]. Despite these promising results, it remains to be determined if all these metabolic benefits can be extrapolated to humans [107]. Additionally, bidirectional interactions between bile acid synthesis and gut microbiota have also been implicated in the regulation of host metabolism [108]. Gut microbiota-derived secondary bile acids act through the nuclear farnesoid X receptor (FXR) and the Takeda G protein-coupled membrane receptor 5 (TGR5) to regulate different peripheral metabolic pathways [109]. Moreover, acting directly in hypothalamic TGR5 [110] and through diverse indirect pathways can also signal to the CNS to regulate food intake [111,112]. In turn, bile acid signaling can influence microbial composition [113]. Interestingly, both gut microbes and their different metabolites can act as modulators of BBB integrity, hence altering the permeability to peripheral hormones and other factors which can regulate brain activity [74].

The intestinal microbiota is one of the main sources of neurotransmitters. Accumulating evidence in pre-clinical studies highlights that, by manipulating the microbial composition of the GI, neurotransmitter levels can be altered and potentially affect both the enteric and central nervous systems [114]. Over 90% of 5-HT, which modulates melanocortin neurons to mediate its anorexigenic effect [115], is produced in the GI tract by enterochromaffin cells (ECs). Remarkably, it has been observed that indigenous microbiota is able to modulate the peripheral levels of 5-HT by increasing its biosynthesis in a postnatally inducible and reversible manner due to an upregulation of tryptophan hydroxylase 1, the rate-limiting enzyme in the biosynthesis of serotonin [116]. These results, observed by Yano et al. in samples from the colon of mice and from a healthy human colon, also highlight that human- and mouse-derived gut microbiota can promote colonic 5-HT production through stimulatory activities of SCFAs on EC cells. Likewise, secondary bile acids can regulate the 5-HT synthesis and its release from EC cells [116,117]. Besides SCFAs, microbial tryptophan catabolites, such as tryptamine, are able to stimulate 5-HT production and to affect cognitive functions and host activities [118,119]. Likewise, other tryptophan catabolites, kynurenine, quinolinate, indole, and indole derivatives, among others, are able to signal specifically to the brain and finally to influence behaviour [120]. Moreover, the deconjugation process of glucuronide-conjugated 5-HT by bacterial enzymes could be one of the mechanisms via which commensal microbiota modulates 5-HT host levels [121].

GABA, the main inhibitory neurotransmitter in the CNS, can also be affected by the GM [62,122,123]. In fact, manipulating microbiota composition by fecal microbiota transplantation may alter GABA levels [124] and have a great impact on hypothalamic feeding regulation [125]. GABAergic neurotransmission in hypothalamic neurocircuits stimulates feeding and its synaptic release by agouti-related protein-expressing neurons in the arcuate nucleus (ARC), required for normal regulation of energy balance [126]. On the other hand, GABA participates in the cognitive choice of selecting the type, quantity and quality of food. In this sense, stimulation of GABA receptors in the ventral pallidum (VP) induces behavioral effects similar to those observed after accumbens dopamine depletion [127]. Hence, besides VTA-Nac dopaminergic pathway, VP plays a crucial role in the processing and achievement of effort-related choice and motivated behaviors [128,129]. Additionally, as has been previously described in this manuscript, PFC plays an important role in cognitive functions related to eating and in the top-down control of this behaviour. Noteworthy, the regulation of GABAergic neurotransmission is critical for a proper inhibition of PFC activity under maladaptive behaviors, since PFC hyperactivation can cause impairment of working memory and other cognitive functions [130]. In this context, it has been confirmed in animal models that the exposure to hypercaloric diets decreased GABA levels in PFC [131], partly due to changes in microbiota [132]. These GABA level disturbances could impair the inhibitory processes and finally, lead to overeating regardless of satiety sensation.

Besides these neurotransmitters, the microbiota has been shown to synthesize and induce the activity of other neurochemical compounds, such as DA and histamine, which are also able to modulate the host mood and behaviour [114,133], to a point some authors are already describing as the “psychobiome” [134,135].

## 4. Food Addiction: A New Mental Disorder?

*“Addiction is defined as a chronic, relapsing brain disease that is characterised by compulsive drug seeking and use, regardless of unhealthy consequences and long-lasting changes in the brain”* [136]. In fact, addiction reduces the control over decision-making skills by inducing changes in PFC neurons and in basal ganglia activities, among other brain structures [137]. This induced neuroplasticity process perturbates the brain reward homeostasis and leads to more habitual and more compulsive drug use/behaviour [138,139].

Almost anything in the human environment can be rewarding, giving it the capacity to become addictive. According to this, food and related eating behaviors, like any other stimulus, can cause an addiction. However, there is still an ongoing debate in the scientific community regarding the “food addiction concept” and this paradigm is recurrently revisited, as usually happens with other diseases and mental disorders [5,6,140,141,142,143,144,145,146,147,148]. Indeed, the concept is not new, and has been in constant evolution through the years [145], with increased interest in recent decades. In this context, major controversy exists because food addiction can be considered as a substance-related disorder (food addiction), or a non-substance-related disorder (eating addiction). Hence, while some authors report that people can be addicted to sugar, salt, additives, and high-fat content [149,150,151,152,153], others argue that a behavioral addictive disorder better describes eating problems [6,154]. Finally, a third position has been taken by other researchers, who consider that food addiction is an unnecessary term that could increase the medicalization of common behaviors [155,156]. In agreement, some authors call attention to the fact that using food addiction as a term might increase stigmatizing attitudes, accordingly having an impact on the treatment of these disorders [157].

Addiction is comprised of three steps: preoccupation/anticipation (craving), binge/intoxication, and withdrawal/negative effect. All these aspects have been confirmed, at least in animal models [5]. However, some authors consider that these steps are not convincingly observed in the context of human food addiction [158]; consequently, they suggest current models of addiction should be re-evaluated. To be noted, since food intake is essential for survival, and eating behaviour is an important social practice [159], these aspects should be contemplated when approaching eating disorders.

Many addiction researchers and clinicians, describe “addiction” as a brain disease [160,161], which can be partly explained by the DA deficiency hypothesis [162]. According to this hypothesis, some subjects will be immersed in abnormal craving behaviors, such as over-consumption of highly rewarding food, to compensate for the DA deficiency [163]. In fact, compulsive-like feeding behaviour in obese rats was linked to the downregulation of striatal dopamine D2 receptors (D2Rs). Likewise, after silencing striatal D2Rs in rats, these animals showed compulsion-like food seeking [164]. Besides DA deficiency, these subjects also present a dysregulation of other neurotransmitters. On the other hand, a new learning model has been proposed recently to explain how addictive behaviors could take place. This model suggests that addictive behaviour is learned and is not the result of pathologic brain signaling [165]. Since our brain is plastic, we can modify our behaviors throughout our lives by learning new habits to adapt to changes in our environment. In fact, as already pointed out by Dr Woods thirty years ago, food itself is necessary but the act of eating is disruptive, accordingly humans have to learn new responses to tolerate it [166]. Regardless of the vision of addiction as a brain disease or as a learning process, both highlight that addiction is a maladaptive behaviour with adverse consequences. Probably, addictive eating behaviors can be explained by a sum of both models, due to a close interaction between brain changes and social conditions. Noteworthy, if our brain can change in a negative manner as observed in addiction, could our brain switch in a healthier way by adopting new habits?

## 5. Interrelationship between Gut Microbiota and Food Addiction

**Figure 3:**
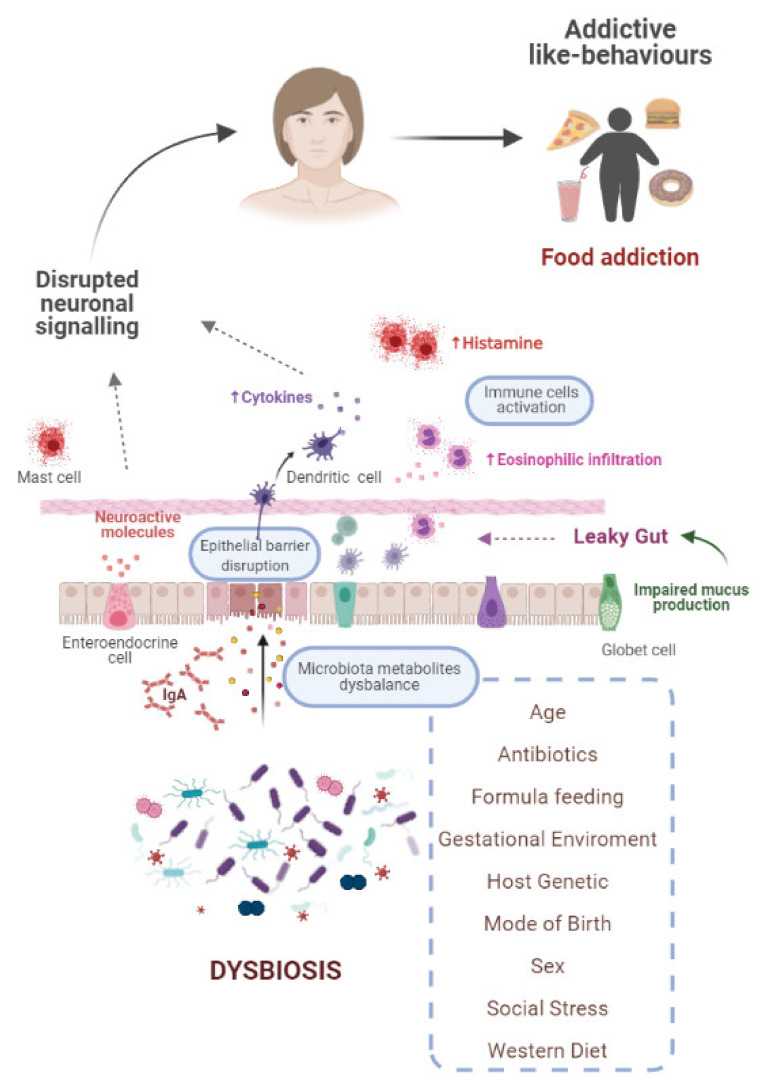
Gut-brain axis and dysbiosis. Possible mechanisms implicated in the development of addictive like-behaviors, such as “food addiction”, as result of a brain disrupted signaling.

A great number of studies have implicated the GM as a key modulator of brain and behaviour, and have shown how bidirectional communication through the GMB axis is essential for the regulation of host metabolism and energy homeostasis [9,167]. However, fewer studies have been conducted to answer how the microbiota might influence addiction-related behaviors, such as “food/eating addiction”. Even though the research to date is not complete, increasing evidence shows how microbiota dysbiosis is implicated in the development of these maladaptive habits [8,10,58,168,169,170,171,172] (Figure 3). Considering this, all factors affecting the “healthy” composition of the GM, including host genetics [173,174], diet [175,176], age [177,178], mode of birth [179,180], and antibiotics intake [181,182], among others, can shape GM and ultimately trigger an abnormal eating behaviour. In fact, recently, Dong et al. have reported that females with obesity and food addiction present a different GM when compared to females without these conditions [183]. Noteworthy, some of these changes can be the result of prenatal factors, similar to the fetal programming described in the context of other mechanisms regulating food intake [184,185]. Different studies conducted in animal models have highlighted that changes in maternal microbiota during pregnancy influence neonatal gut microbiome and have permanent effects on offspring behaviour. Probably, the role of maternal diet during the gestation period has been the most commonly studied factor. Both human and non-human studies evidence that there is a close association between maternal diet, maternal microbiome and infant microbiome. Hence, nutrition during pregnancy modulates maternal microbiota, and this could lead to a negative impact on offspring brain development due to infant dysbiosis [186,187]. Accordingly, all gestational complications affecting maternal microbiomes can potentially cause neurodevelopmental disorders and exert long-lasting effects on offspring behaviour [188]. Interestingly, Jasarevic et al. demonstrate that changes in the composition of maternal vaginal microbiota due to a stressful situation can influence offspring gut and hypothalamus increasing the risk of neuro-disorders [189].

Besides these pre- and neonatal influences, the microbiota is highly susceptible to change in early life. In this context, breastfeeding or formula feeding might have a role in future behaviour by modulating GM infant composition differently [190]. Breastfeeding has been positively associated with early brain development and cognitive function as observed by Liu et al. In this study, conducted in infant rhesus macaques, authors describe significant brain structural differences between breastfed, which promoted maturation of cortical areas, and formula-fed animals [191]. Interestingly, studies conducted on children have also shown that breastfed subjects have healthier dietary patterns in life [192]. Considering all these data, we could conclude that any perturbation of host-microbiota during a critical window period has persisting consequences in host-metabolism, as observed in mice [193]. On the other hand, during this period, the microbiome has an important role in programming the HPA axis for stress response, as already mentioned [77]. In agreement, chronic early-life stress, such as maternal separation, in mice leads to intestinal dysbiosis, which determines abnormal behaviors [194]. These aberrant effects could be mediated by disruption in the myelination and brain development processes, since as observed in pre-clinical models early-life microbiome is implicated in the myelination of PFC and synapse strial function, both areas crucial for proper eating behaviour [195,196]. However, since most research has been conducted in animal models, more data from human studies are needed to decode if these perturbations might increase the risk of developing eating disorders in adulthood.

In addition to early-life influences, dysbiosis can result from exposure to other environmental factors throughout life, including diet, toxins, drugs, and pathogens, as well as social stress. Depending on the predominant macronutrients in the diet, different species of microorganism will be stimulated in the GM. In this sense, the Western diet, rich in fat and simple carbohydrates with low levels of fiber, results in less diversity of the intestinal microbiota. Moreover, Sonnenburg et al. showed in humanized mice that the loss of diversity in the composition of GM after western diet (WD) intake was magnified over several successive generations [197]. This shift to an “unhealthy” microbiota composition induced by the WD influences brain function and induces addictive-like eating behaviors [198]. Studies conducted both in humans and animal models confirm that consumption of highly palatable food and ultra-processed food typical of WD is closely related to the development of these maladaptive habits [199,200,201,202]. Based on these data, the vicious cycle hypothesis has been proposed. Accordingly, the diet provides the substrate for the GM, which modulates appetite by signaling the brain, and finally CNS mediates the preference for specific foods, and the cycle starts once more [198].

One of the potential mechanisms proposed to explain brain alterations has been the “leaky gut” [203]. An imbalanced GM induces changes in gut permeability, hence increasing the translocation of microbial metabolites, known as endotoxemia, from the lumen of the GI tract to the adjacent tissues and finally to the systemic circulation. These metabolites can signal the brain to modulate host behaviour, which can explain why many CNS disorders have been linked to a compromised gut barrier [204]. Noteworthy, a damaged intestinal barrier results from some of the factors already commented, such as WD and stress, among others.

Importantly, many microbiota-associated changes occur in a sex/gender-dependent manner and these differences can influence the brain and behaviour [205,206,207]. In this context, women report higher food addiction behaviors, cravings and reward sensitivity than men [208,209]. Accordingly, recently, sex addiction-phenotype and related behaviors have been associated with the microbiome in a rodent model [210]. All these data highlight that that the close interaction between microbiota and sex/gender should be considered in future studies.

## 6. Conclusions

How the gut microbiota signals the brain to regulate eating behaviour has been the subject of significant research over the past decade and there is no doubt that the gut microbiome plays a crucial role in host metabolism and eating behaviour. However, although the evidence suggests that targeting the microbiota could serve as a promising therapeutic option for some mental disorders, such as addiction-like behaviors, to date the majority of data on the microbiota–gut–brain axis has been obtained from studies using animal model systems. Animal studies are basic in understanding some molecular mechanisms, but these potential clinical implications should be assessed in-depth in clinical models. Despite these limitations, one of the most hopeful treatments for modifying the GM to improve eating disorders would be the use of probiotics and prebiotics, not only to treat but also to prevent the unhealthy microbiome disbalance. Our microbial system is complex, but it has been already mentioned throughout this manuscript that it directly impacts on our cognitive function and mood. Therefore, every positive impact on our microbiota due to probiotics intake should be considered in the context of eating disorders.

On the other hand, irrespective of the “food addiction” concept, researchers from different fields including neuroscience, neuroendocrinology, psychology, and many others should work together and go forward to fill existing knowledge gaps and to provide a valid framework for the prevention and treatment of addictive eating behaviors. In this context and considering the state of the art, both pharmacological treatments and cognitive behavioral interventions should be considered. Finally, although evidence suggests that obesity, binge eating disorder (BED) and addictive eating behaviour share the same reward pathways, and furthermore some of their symptoms can overlap, they are considered as independent pathologies. Hence, even if obesity, eating disorders, addictive eating behaviour and other addictions could be intimately connected, they should be approached differently.
